# Supplementary material for: Ribose-cysteine protects against the development of atherosclerosis in apoE-deficient mice
Source: PLoS One. 2020 Feb 21;15(2):e0228415. doi: 10.1371/journal.pone.0228415 (PMC7034848; doi:10.1371/journal.pone.0228415)
Supplement: S1 Fig — The raw response data was normalised to an internal standard (13C6 sorbitol) to account for instrumental variations and the normalised response were plotted over the incubation time. The stability experiments were performed at room temperature. (DOCX) [file pone.0228415.s001.docx]

**S1 Fig**


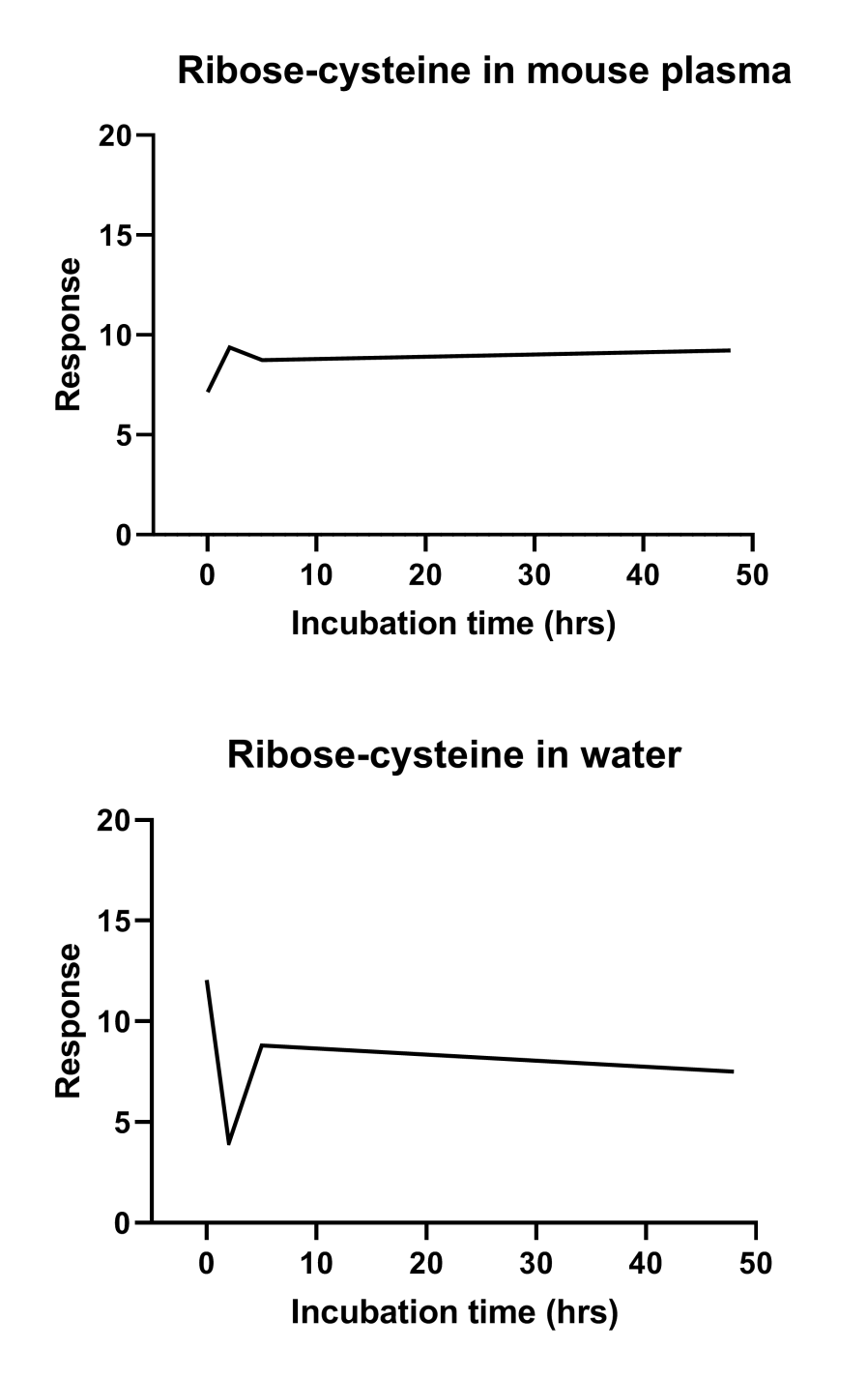


**S1 Fig. Stability of ribose-cysteine (20 µM) in plasma or water up to 48 hours.** The raw response data was normalised to an internal standard (^13^C_6_ sorbitol) to account for instrumental variations and the normalised response were plotted over the incubation time. The stability experiments were performed at room temperature.
