# Supplementary material for: Ribose-cysteine protects against the development of atherosclerosis in apoE-deficient mice
Source: PLoS One. 2020 Feb 21;15(2):e0228415. doi: 10.1371/journal.pone.0228415 (PMC7034848; doi:10.1371/journal.pone.0228415)
Supplement: S2 Fig — The data is presented as mean fold difference in peak areas ±standard deviation after median normalisation. Student t-test with Benjamini-hochberg correction was used for statistical analysis using R (v3.3.1). (DOCX) [file pone.0228415.s002.docx]

**S2 Fig**


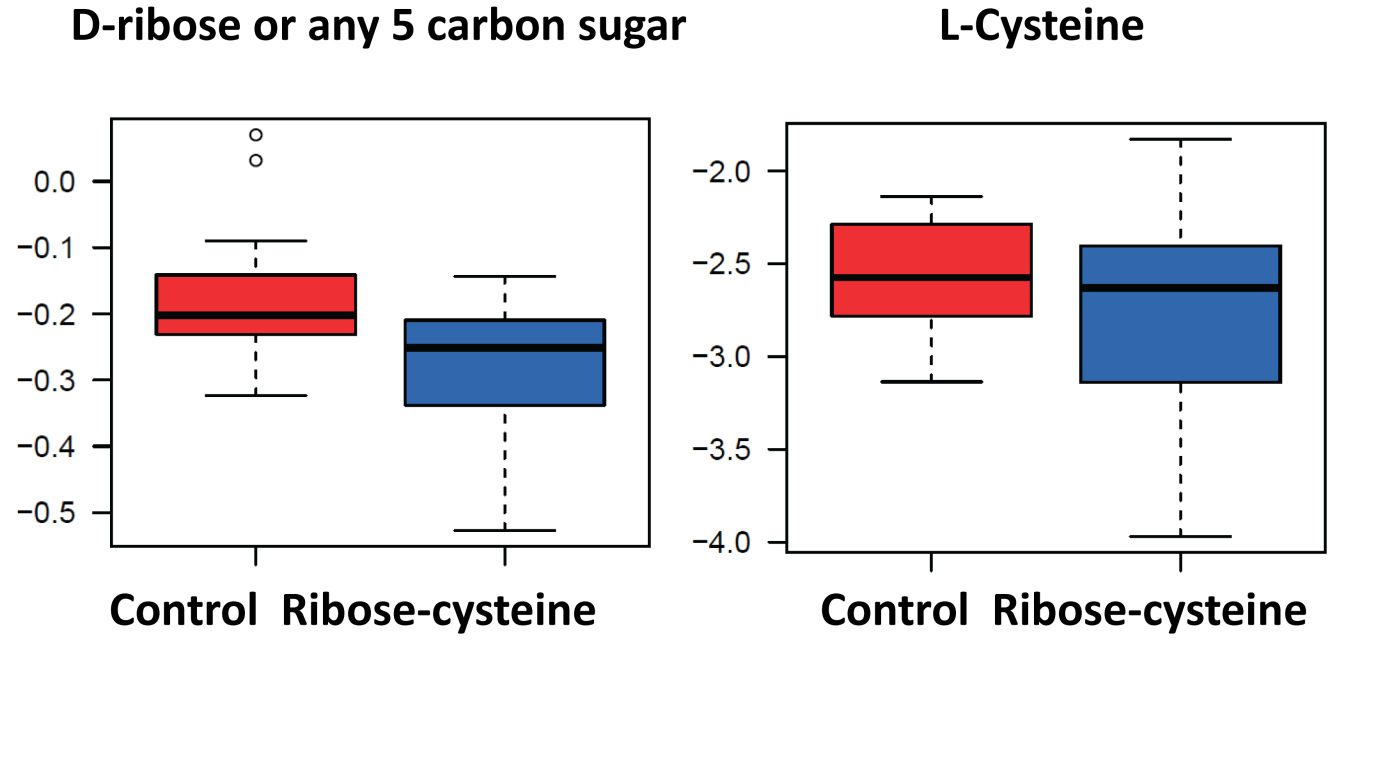


**S2 Fig. Ribose-cysteine-related metabolites detected in plasma and compared between the two groups.** The data is presented as mean fold difference in peak areas ±standard deviation after median normalisation. Student t-test with Benjamini-hochberg correction was used for statistical analysis using R (v3.3.1).
