# Supplementary material for: Ribose-cysteine protects against the development of atherosclerosis in apoE-deficient mice
Source: PLoS One. 2020 Feb 21;15(2):e0228415. doi: 10.1371/journal.pone.0228415 (PMC7034848; doi:10.1371/journal.pone.0228415)
Supplement: S3 Fig — The LDLR (A) and HMGCoA reductase (B) proteins were analysed by western blotting of liver homogenates (40μg) from treated and control mice. Representative blots for 13 control and 14 treated mice are shown. (DOCX) [file pone.0228415.s003.docx]

**S3 Fig**

**
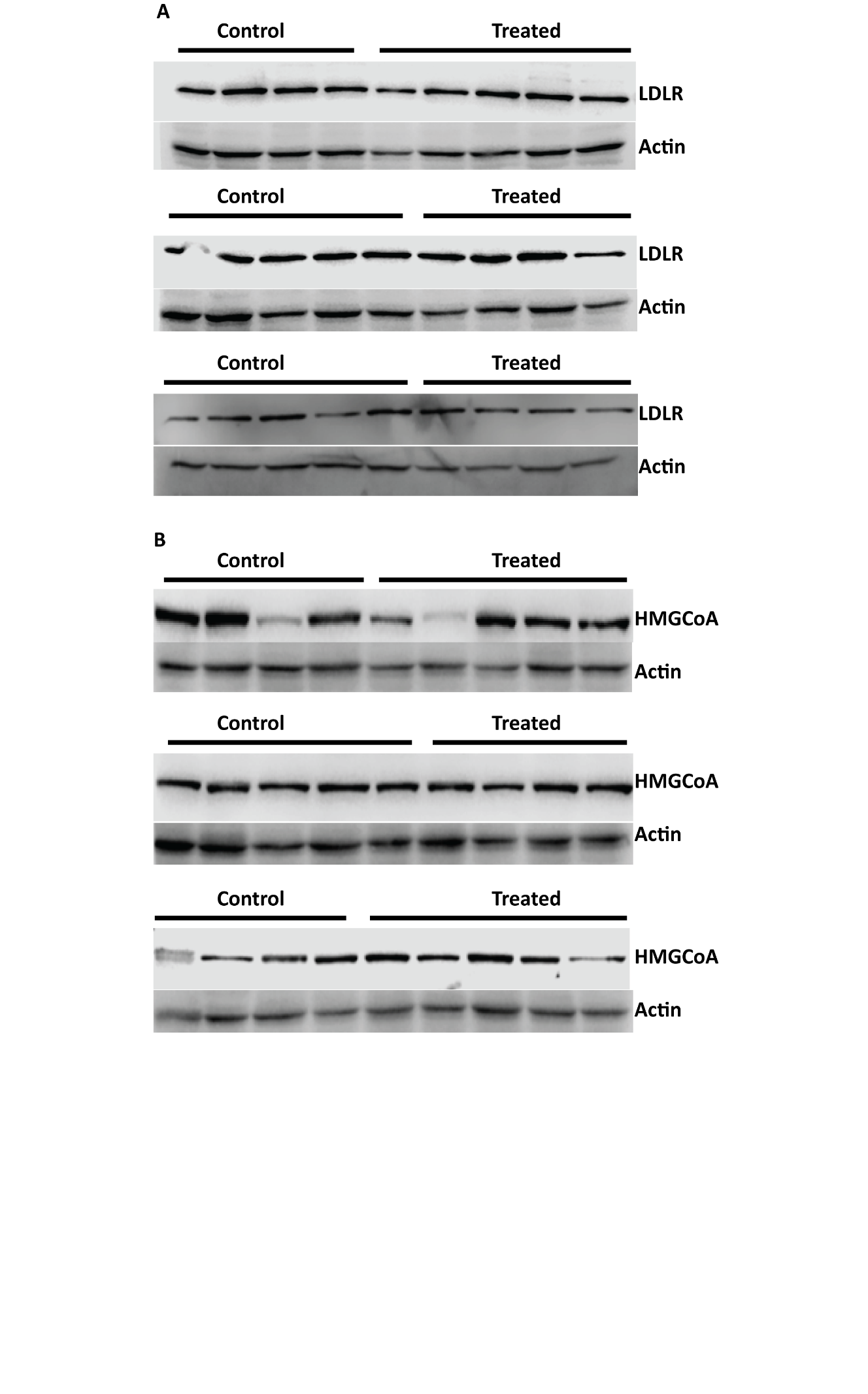
**

**S3 Fig. Ribose-cysteine has no effect on levels of the low-density lipoprotein receptor (LDLR) and the HMGCoA reductase protein in the liver.** The LDLR (A) and HMGCoA reductase (B) proteins were analysed by western blotting of liver homogenates (40µg) from treated and control mice. Representative blots for 14 control and 13 treated mice are shown.
