# Supplementary material for: Ribose-cysteine protects against the development of atherosclerosis in apoE-deficient mice
Source: PLoS One. 2020 Feb 21;15(2):e0228415. doi: 10.1371/journal.pone.0228415 (PMC7034848; doi:10.1371/journal.pone.0228415)
Supplement: S4 Fig — The SR-B1 protein was analysed by western blotting of liver homogenates (40μg) from treated and control mice. Representative blots for 15 control and 15 treated mice are shown. Fold difference of relative SR-B1 protein, **p<0.01, Kolmogorov-Smirnov test was used for statistical analysis. Error bars indicate means ± SEM. (DOCX) [file pone.0228415.s004.docx]

**S4 Fig**


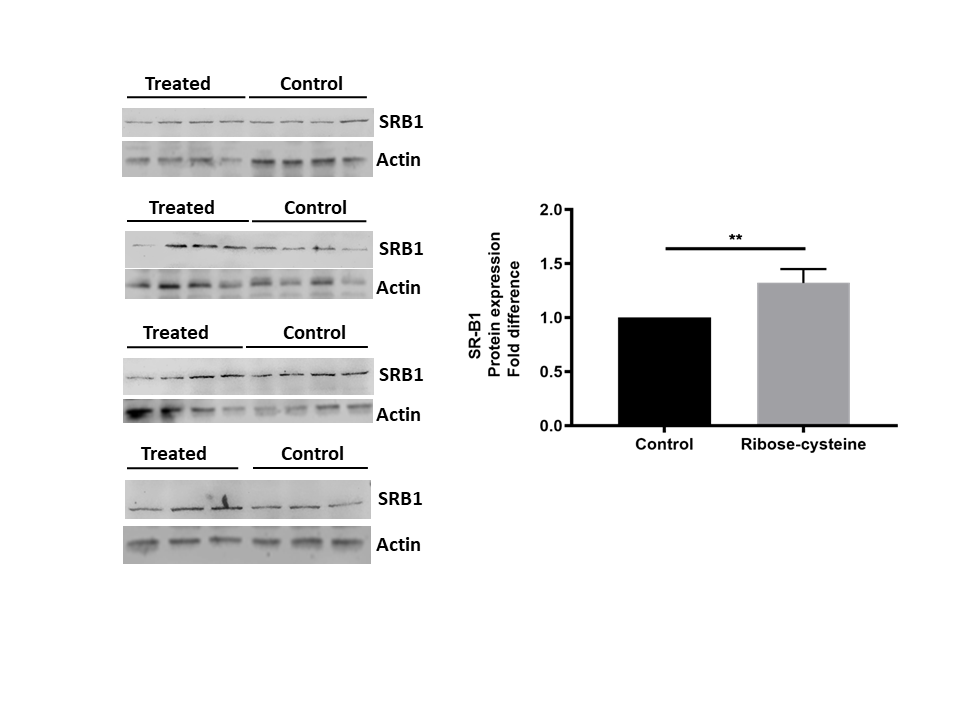


**S4 Fig. Ribose-cysteine increases SR-B1 protein expression in the liver.** The SR-B1 protein was analysed by western blotting of liver homogenates (40µg) from treated and control mice. Representative blots for 15 control and 15 treated mice are shown. Fold difference of relative SR-B1 protein, **p<0.01, Kolmogorov-Smirnov test was used for statistical analysis. Error bars indicate means ± SEM.
