# Supplementary material for: Ribose-cysteine protects against the development of atherosclerosis in apoE-deficient mice
Source: PLoS One. 2020 Feb 21;15(2):e0228415. doi: 10.1371/journal.pone.0228415 (PMC7034848; doi:10.1371/journal.pone.0228415)
Supplement: S2 File — (PPTX) [file pone.0228415.s006.pptx]

## Slide 1
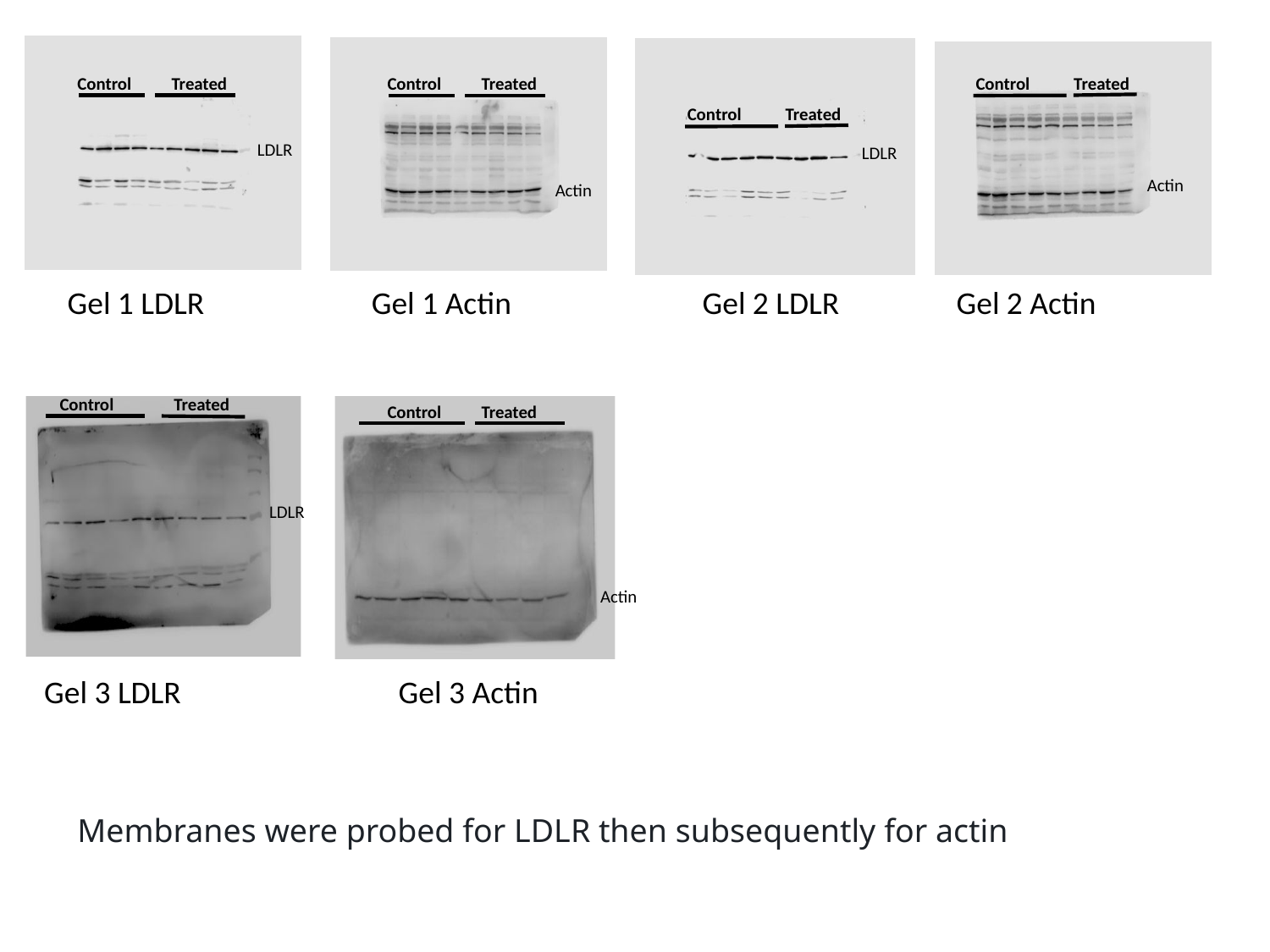

Control Treated
Control Treated
Control Treated
Control Treated
LDLR
LDLR
Actin
Actin
Gel 1 LDLR 	 Gel 1 Actin		Gel 2 LDLR	Gel 2 Actin
Control Treated
Control Treated
LDLR
Actin
Gel 3 LDLR 	 Gel 3 Actin
Membranes were probed for LDLR then subsequently for actin

## Slide 2
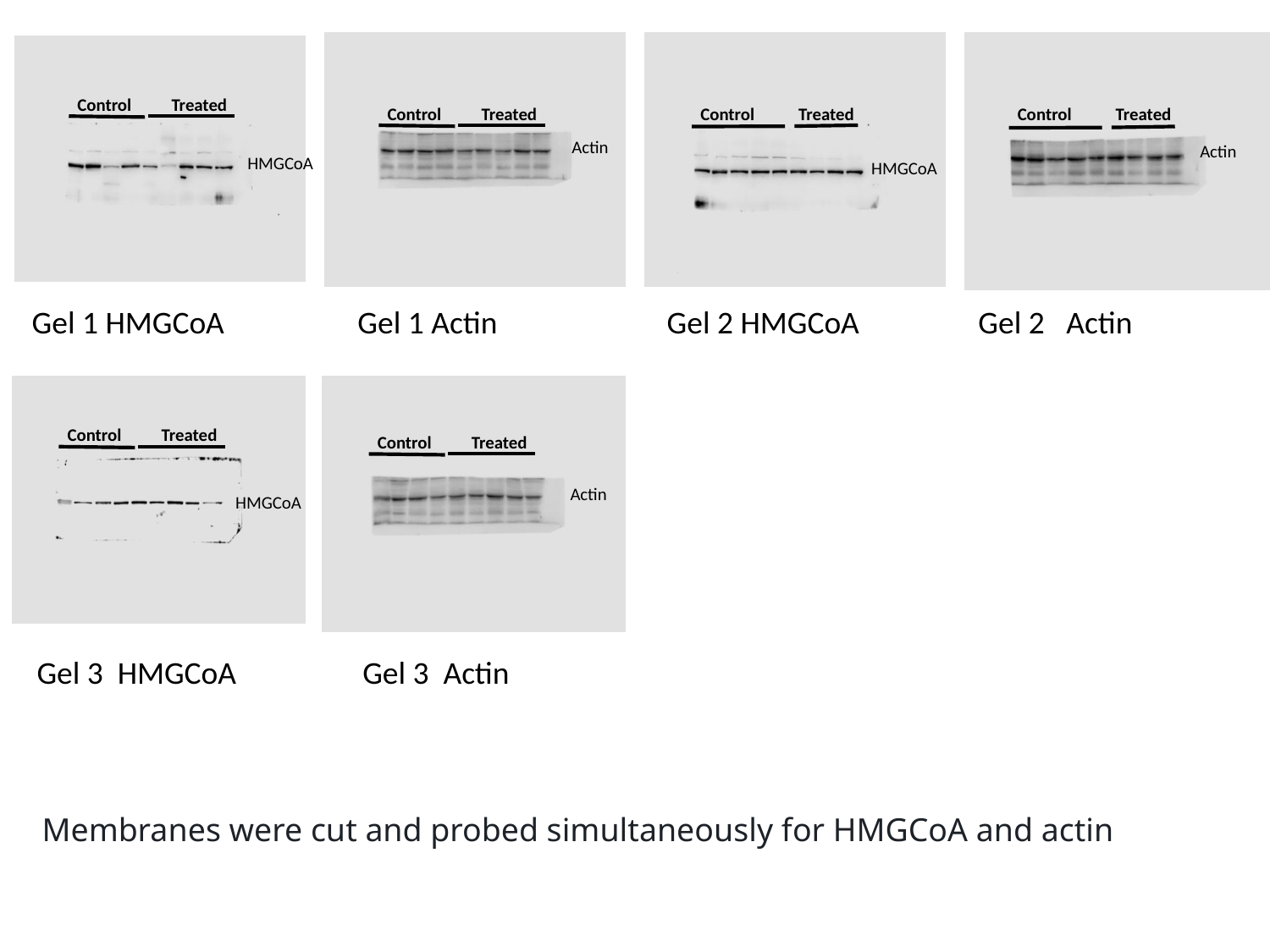

Control Treated
Control Treated
Control Treated
Control Treated
Actin
Actin
HMGCoA
HMGCoA
Gel 1 HMGCoA 	 Gel 1 Actin		Gel 2 HMGCoA	 Gel 2 Actin
Control Treated
Control Treated
Actin
HMGCoA
Gel 3 HMGCoA 	 Gel 3 Actin
Membranes were cut and probed simultaneously for HMGCoA and actin

## Slide 3
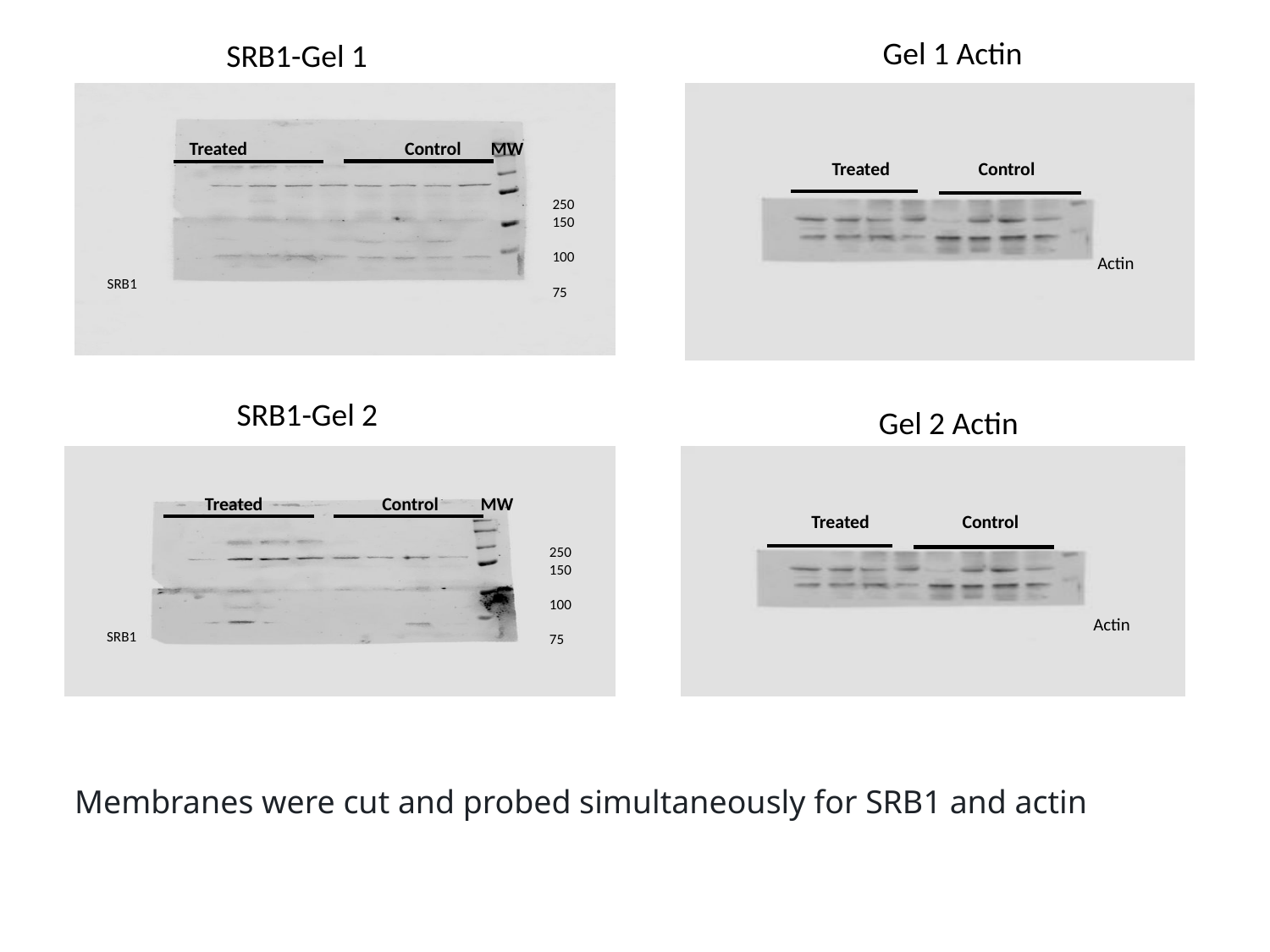

Gel 1 Actin
SRB1-Gel 1
 Treated 	 Control MW
 Treated Control
250
150
100
75
Actin
SRB1
SRB1-Gel 2
Gel 2 Actin
 Treated 	 Control MW
 Treated Control
250
150
100
75
Actin
SRB1
Membranes were cut and probed simultaneously for SRB1 and actin

## Slide 4
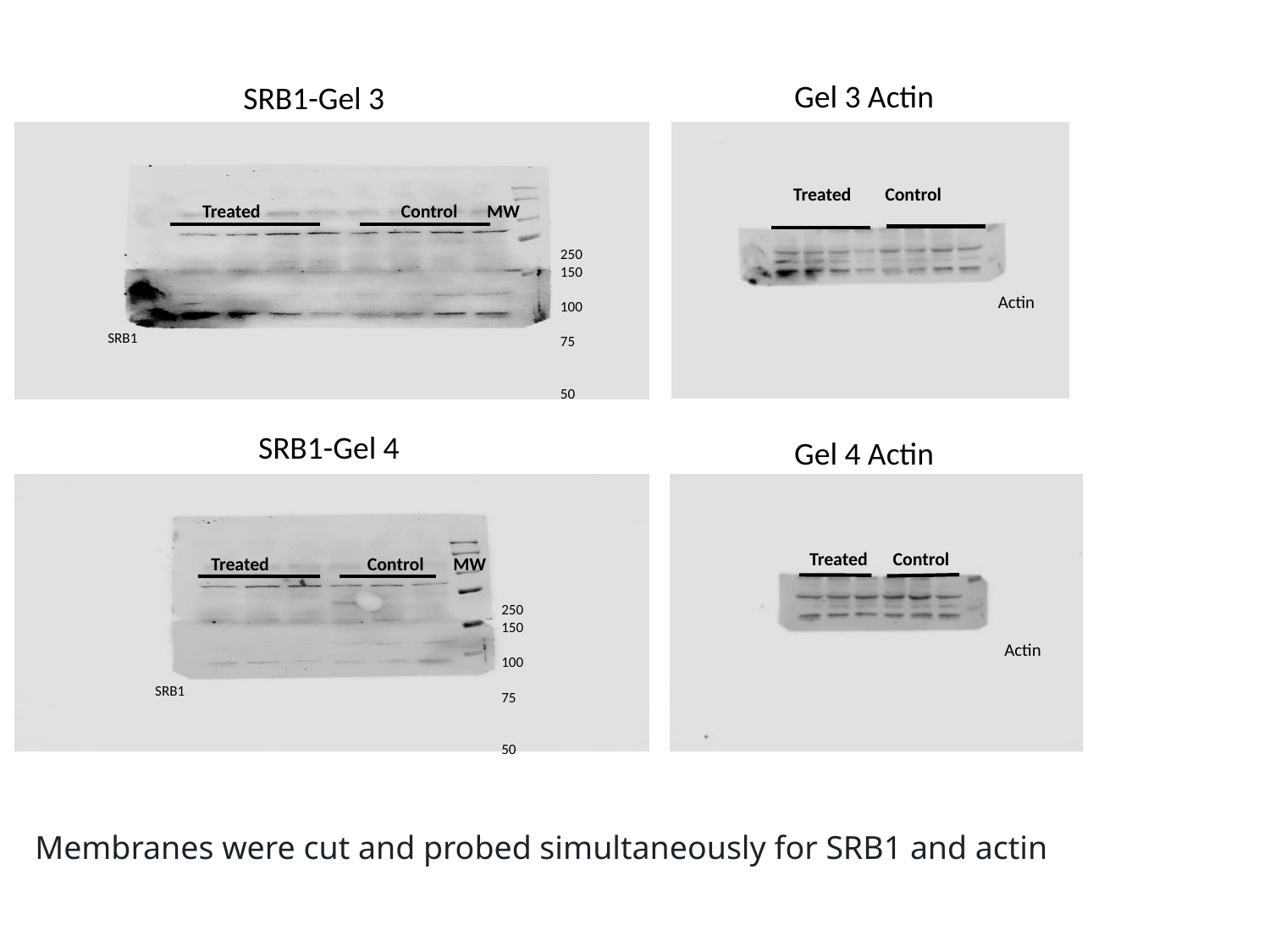

Gel 3 Actin
SRB1-Gel 3
 Treated 	 Control MW
250
150
100
75
50
SRB1
 Treated Control
Actin
SRB1-Gel 4
 Treated 	 Control MW
250
150
100
75
50
SRB1
Gel 4 Actin
 Treated Control
Actin
Membranes were cut and probed simultaneously for SRB1 and actin
