# Supplementary material for: Ribose-cysteine protects against the development of atherosclerosis in apoE-deficient mice
Source: PLoS One. 2020 Feb 21;15(2):e0228415. doi: 10.1371/journal.pone.0228415 (PMC7034848; doi:10.1371/journal.pone.0228415)
Supplement: S3 File — (PPTX) [file pone.0228415.s007.pptx]

## Slide 1
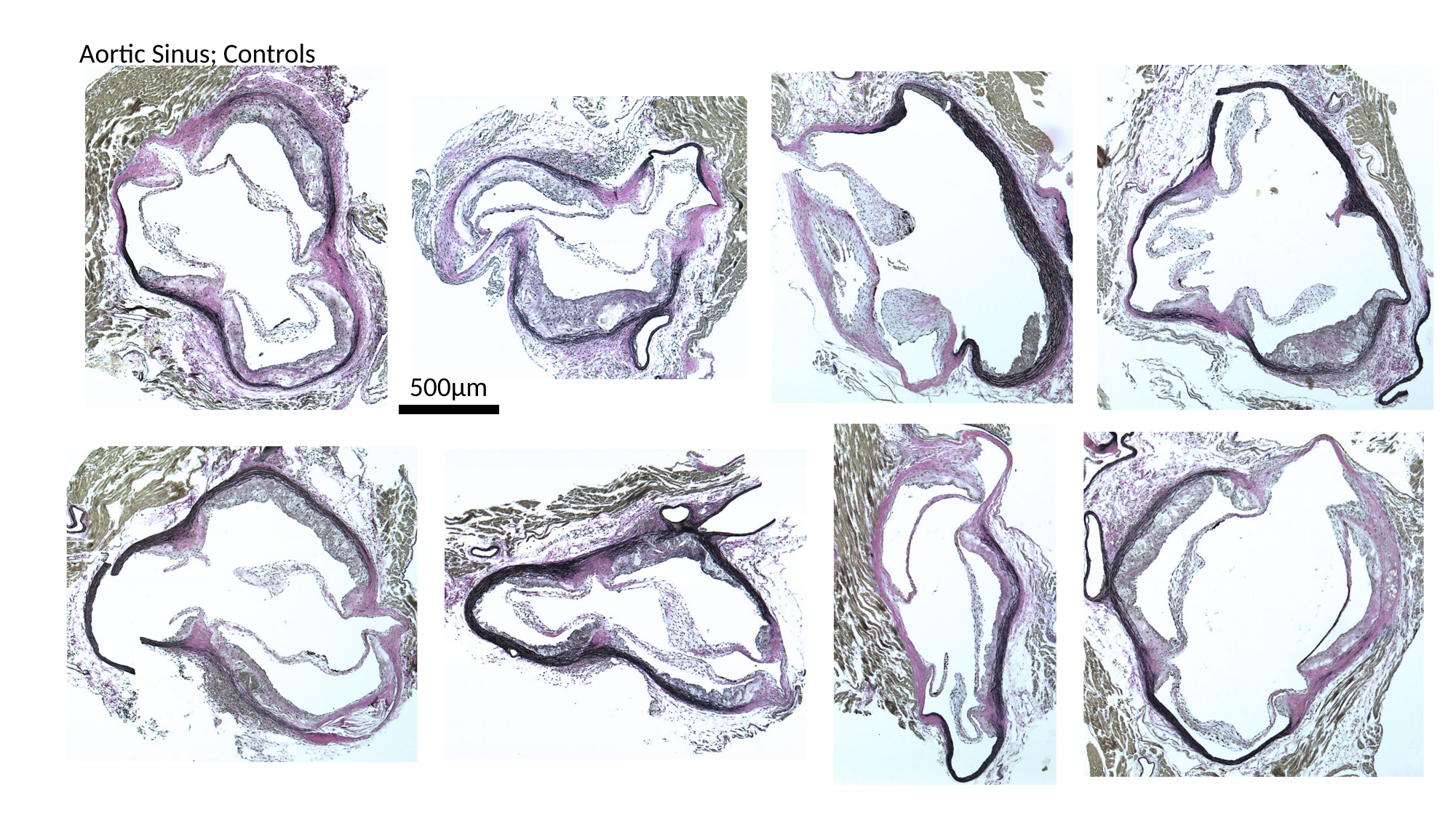

Aortic Sinus; Controls
500µm

## Slide 2
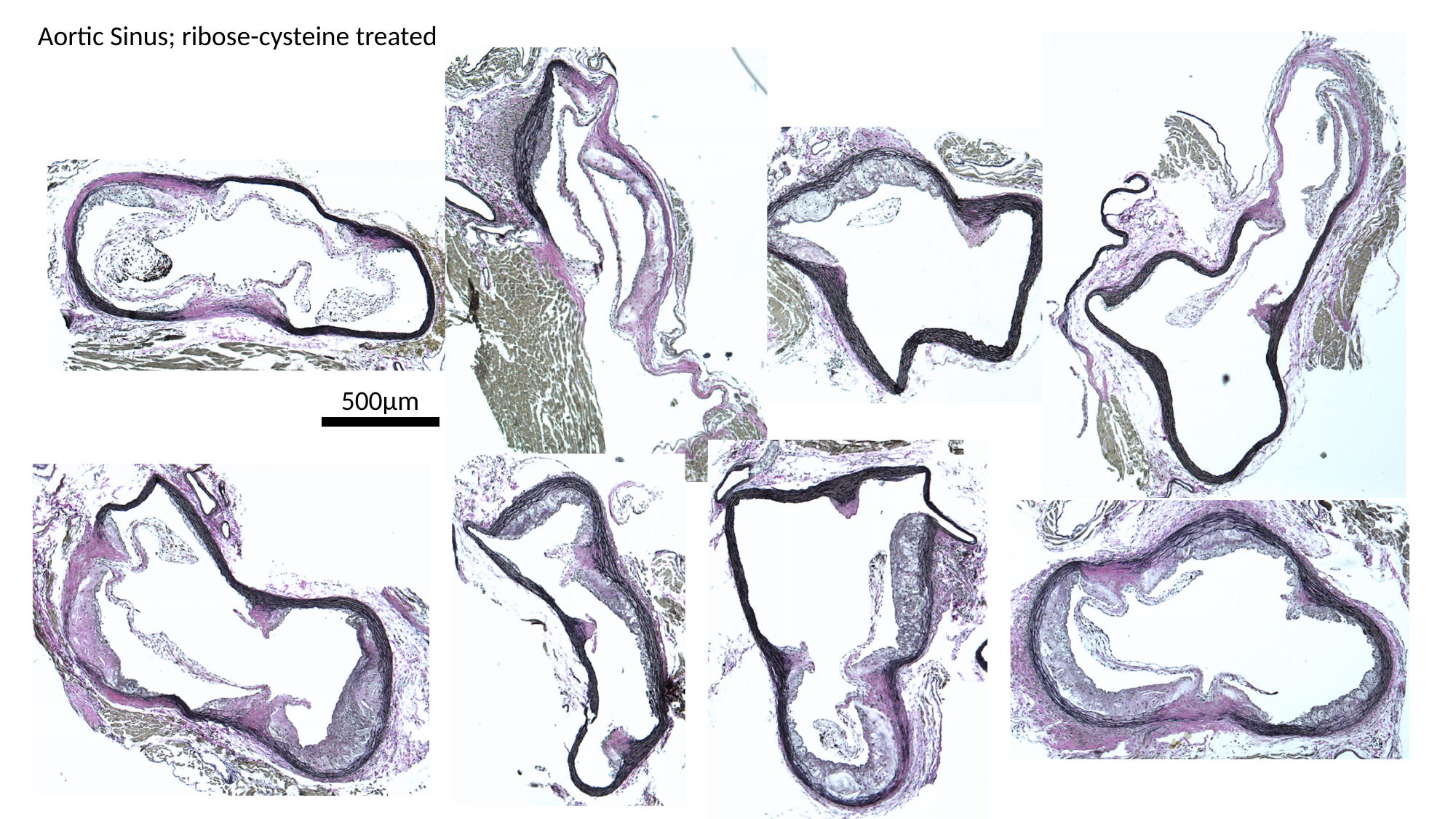

Aortic Sinus; ribose-cysteine treated
500µm

## Slide 3
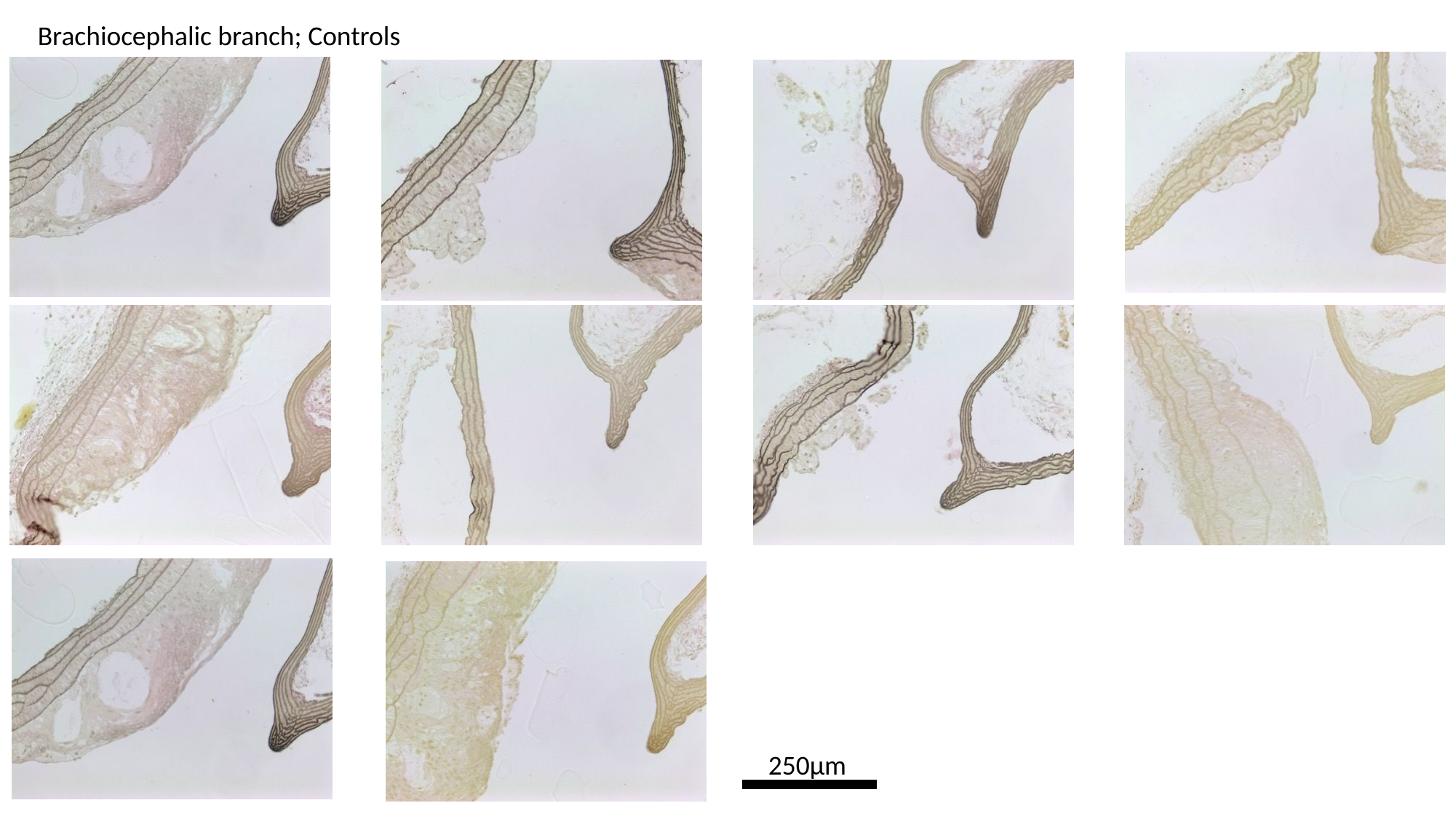

Brachiocephalic branch; Controls
250µm

## Slide 4
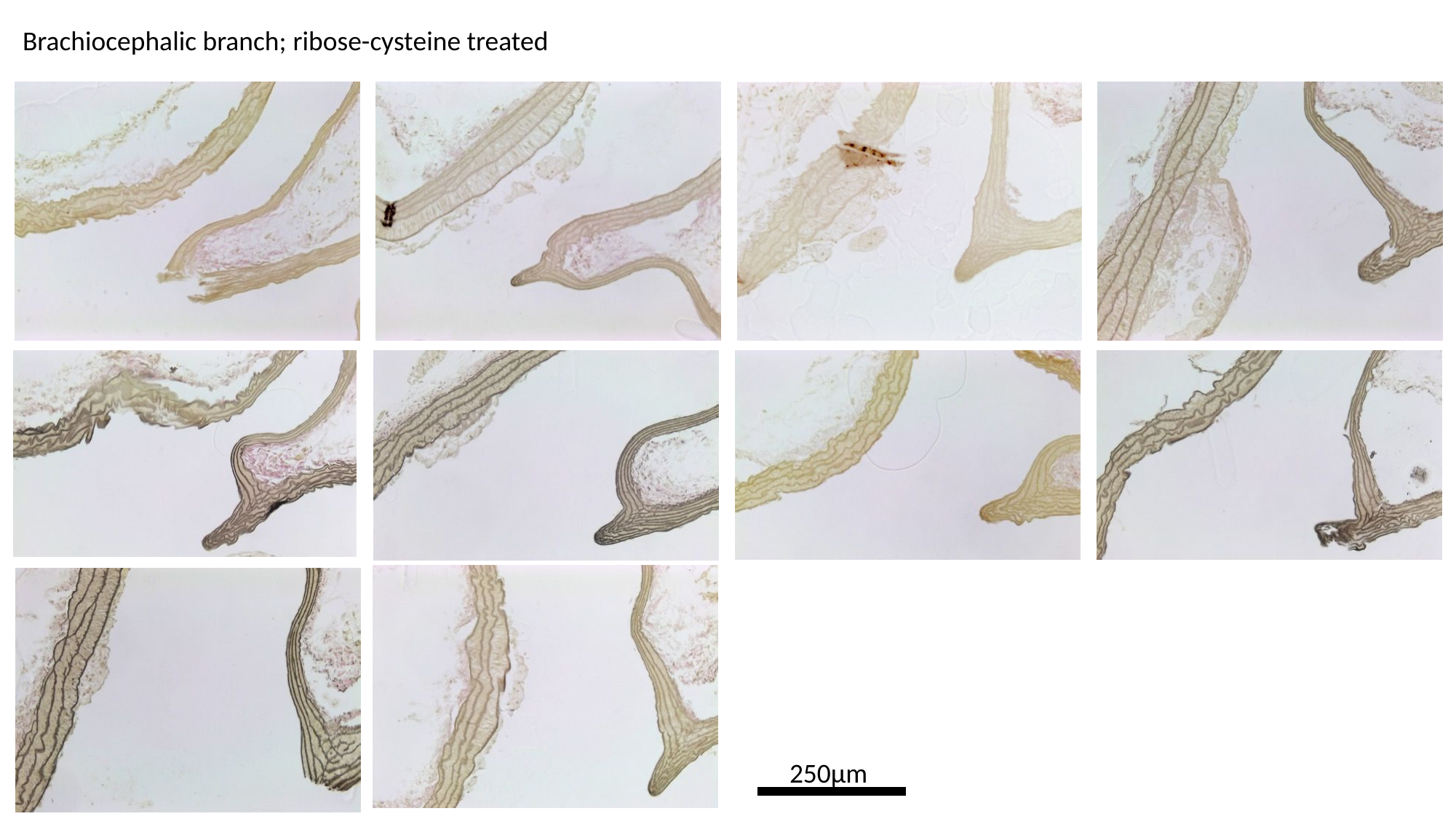

Brachiocephalic branch; ribose-cysteine treated
250µm
